# Cell Cycle Status Influences Resistance to Apoptosis Induced by Oxidative Stress in Human Breast Cancer Cells, Which Is Accompanied by Modulation of Autophagy

**DOI:** 10.3390/cimb45080399

**Published:** 2023-07-29

**Authors:** Magdalena Kluska, Agnieszka Wanda Piastowska-Ciesielska, Paulina Tokarz

**Affiliations:** 1 Department of Molecular Genetics, University of Lodz, Pomorska 141/143, 90-236 Lodz, Poland; 2 Department of Cell Cultures and Genomic Analysis, Medical University of Lodz, Zeligowskiego 7/9, 90-752 Lodz, Poland; agnieszka.piastowska@umed.lodz.pl

**Keywords:** autophagy, breast cancer, cell cycle, DNA damage response, oestrogen, oxidative stress

## Abstract

Cancer cells are characterised by uncontrolled cell proliferation; however, some of them can temporarily arrest their cell cycle at the G0 or G1 phase, which could contribute to tumour heterogeneity and drug resistance. The cell cycle status plays a critical role in chemosensitivity; however, the influence of G0- and G1-arrest has not been elucidated. To study the cell cycle arrest-mediated resistance, we used MCF-7 cells and generated three populations of cells: (1) cells arrested in the G0-like phase, (2) cells that resumed the cell cycle after the G0-like phase and (3) cells arrested in early G1 with a history of G0-like arrest. We observed that both the G0-like- and the G1-arrested cells acquired resistance to apoptosis induced by oxidative stress, accompanied by a decreased intracellular reactive oxygen species and DNA damage. This effect was associated with increased autophagy, likely facilitating their survival at DNA damage insult. The cell cycle reinitiation restored a sensitivity to oxidative stress typical for cells with a non-modulated cell cycle, with a concomitant decrease in autophagy. Our results support the need for further research on the resistance of G0- and G1-arrested cancer cells to DNA-damaging agents and present autophagy as a candidate for targeting in anticancer treatment.

## 1. Introduction

Breast cancer (BC) is a dynamic and heterogeneous disease manifesting in a distinction of clinically relevant tumour-cell subpopulations [1,2]. BC intertumoral and intratumoral heterogeneity explains clinical differences in disease progression and chemotherapy responses. The intratumor heterogeneity emerges from both the tumour microenvironment and substantial variation in cancer cells at the genetic, epigenetic and phenotypic levels [1,3,4,5]. Variability in phenotypic states within an individual tumour translates into cancer cell populations exhibiting significant differences in functional properties, including their ability to proliferate, invade and migrate, their stemness and their intrinsic cell plasticity, which influence their behaviour. However, the intratumor heterogeneity in the context of the cell cycle has been underestimated. The distribution of cell cycle phases among cancer cells within a tumour is heterogeneous, as demonstrated at the single-cell level in triple-negative BC (TNBC) tumours [6]. Cancer cells in an established tumour are predominantly in the G0/G1 phase and are resistant to cytotoxic chemotherapy, as demonstrated in stomach adenocarcinoma cells [7]. However, most cancer cells located at the surface of the tumour are proliferative as they consist of mostly (70~80%) cells in S/G2/M.

Cancer cells can arrest their cell cycle in the G0 or G1 phase due to intratumoral contact inhibition, their physiology—in the case of cancer stem and stem-like cells—or cancer cell invasion [8,9]. From a functional perspective, G0- and G1-arrested cancer cells share some features of cancer stem cells, such as chemoresistance (including insensitivity toward S-phase and M-phase drugs) and a high clonogenic potential [10,11,12]. The survival, migration, and invasion of the G0- and G1-arrested BC cells are highly dependent on autophagy [12,13]. The physiological role of autophagy is the maintenance of cell homeostasis by recycling damaged proteins and/or organelles functioning as a survival mechanism. However, it may also act as a cell death pathway and function as a tumour-suppressive mechanism [13].

**Figure 1:**
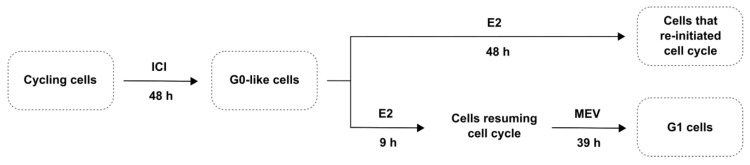
Study design. Logarithmically growing MCF-7 (cycling) cells were arrested in the G0-like phase by ICI 182780 (ICI; 10 nM) for 48 h. Cell cycle reinitiation was induced by 17β-estradiol (E2, 100 nM) for 48 h (re-cycling cells). For the subsequent arrest of the cells in the G1 phase, the cells were treated with mevinolin (MEV; 40 µM) for 39 h immediately after G0 → G1 transition.

Hence, the G0- and G1-arrested cancer cells can persist chemotherapy and re-enter the cell cycle in favourable conditions, manifesting in tumour recurrence [12]. The eradication of all cancer subclones, including those present at a very low frequency, is critical for the ultimate clinical outcome [10]. The potential contribution of the G0- and G1-arrested cancer cells in tumour recurrence prompted us to investigate the molecular mechanisms underlying the BC cells’ sensitivity to DNA-damaging agents in a cell cycle-dependent context. In the present work, we compared some aspects of DNA damage response (DDR) in cycling and arrested cells and cells that resumed the cycle after arrest. We distinguished three populations of cells: (1) the cells arrested in the G0-like phase, (2) the cells which reinitiated the cell cycle, and (3) the cell arrest in early G1 with a history of previous G0-like arrest (which will be further referred to as G1-arrested cells) (Figure 1). We denote cells as G0-like cells but not ‘quiescent’ as initially named, as we believe ‘quiescence’ is reserved for stem cells. We compared DDR, including apoptosis, autophagy, and cell cycle distribution, in these cells.

## 2. Materials and Methods

### 2.1. Cell Line

Human breast adenocarcinoma cell line MCF-7 (ATCC-HTB-22) was purchased from American Type Culture Collection (ATCC™, Manassas, VA, USA). The cells were cultured in RPMI medium with 25 mM HEPES (Lonza, Basel, Switzerland) containing 10% FBS (Biowest, Nuaillé, France), 10 µg/mL insulin (I9278, Sigma-Aldrich, Poznan, Poland), 2 mM L-glutamine, 100 units/mL penicillin and 100 μg/mL streptomycin (Lonza). The cell line was incubated in a humidified atmosphere of 5% CO_2_ and 95% air at 37 °C. The cultured cells from passages 9–20 were used for the experiments.

### 2.2. Cell Treatment

MCF-7 cells were seeded onto tissue-culture plates in the medium and allowed to attach for 24 h. The following day the cells were washed with PBS and incubated with 10 nM ICI 182780/fulvestrant (ICI, I4409, Sigma-Aldrich) for 48 h in the medium without insulin to induce G0-like arrest [14]. Then, the cells were washed with PBS and treated for 48 h with 100 nM 17β-estradiol (E2, E2758, Sigma-Aldrich) in the presence of 10 μg/mL insulin to reinitiate the cell cycle [15,16,17]. For the induction of early G1 arrest, the cells were first treated with ICI for 48 h, followed by 9 h treatment with E2 and a final treatment with 40 μM mevinolin/lovastatin (MEV, M2147, Sigma-Aldrich) for 39 h [18]. After incubation with different ICI/E2/MEV combinations, the cells were treated with hydrogen peroxide (H_2_O_2_) (216763, Sigma-Aldrich) to induce oxidative stress. 

### 2.3. Mevinolin Preparation

MEV was prepared as described elsewhere [19]. Shortly, 12.5 mg MEV in the inactive lactone form was dissolved in 250 μL warmed ethanol (56 °C). Then, 195.5 μL 1 M NaOH and 2.25 mL H_2_O were added, and the mixture was stirred for 1 h at room temperature to allow the conversion of MEV to its sodium salt. pH was normalised to 7.5 with 1 M HCl (ca. 190 μL), and H_2_O was added to obtain a 10 mM solution of active dihydroxy-open acid. The solution was filtered through 0.2 μm pore membranes, aliquoted and stored at –20 °C until use.

### 2.4. Cell Viability

Cell viability was assayed with Muse Count and Viability Kit (Millipore, Hayward, CA, USA), which differentially stains viable and dead cells based on their permeability to two DNA binding dyes. The samples were measured on the Muse Cell Analyzer (Millipore) with MuseSoft 1.4.0.0 software.

### 2.5. Expression of *MKI67* and CCND1

Total RNA was isolated using Extractme Total RNA Kit (Blirt S.A., Gdansk, Poland) according to the manufacturer’s protocol. The quantity and quality of RNA were assessed with spectrophotometer Synergy HT (BioTek Instruments, Winooski, VT, USA). The quantitative real-time PCR reaction was carried out using 2× SensiFAST Probe No-ROX One-Step (Bioline Reagents, London, UK) kit, and real-time gene expression analysis of target genes (*MKI67* and *CCND1*) was performed using TaqMan^®^ Gene Expression Assay (Life Technologies, Foster City, CA, USA). The *GAPDH* (glyceraldehyde 3-phosphate dehydrogenase) gene was used as a reference. The assay numbers for these genes were as follows: Hs01032443_m1 for *MKI67*, Hs00765553_m1 for *CCND1* and Hs_99999905_m1 for *GAPDH*. Each PCR reaction was performed in a 10 μL sample that included 5 μL of 2× SensiFAST Probe No-ROX One-Step Mix (Bioline), 0.1 μL of reverse transcriptase (Bioline), 0.2 μL of RiboSafe RNAse inhibitor (Bioline), 4.2 μL of water-diluted cDNA template (100 ng), and 0.5 μL of TaqMan^®^ Gene Expression Assay, which consisted of a pair of unlabelled PCR primers and a TaqMan probe with a FAM™. The quantitative real-time PCR reaction was carried out using the CFX96 Touch™ Real-Time PCR Detection System (Bio-Rad, Hercules, CA, USA) in the following conditions: reverse transcription for 20 min at 45 °C, denaturation for 2 min at 95 °C followed by 40 cycles of 5 s at 95 °C, 1 min annealing and extension at 60 °C. Relative RNA quantification was performed using the 2^−ΔΔCt^ method [20].

### 2.6. Intracellular ROS Production

Cells after treatment with ICI/E2/MEV were washed twice with HBSS containing Ca^2+^ and Mg^2+^ and stained with 5 µM 2′,7′-dichlorodihydrofluorescein diacetate (H_2_DCF-DA) (Life Technologies) in HBSS containing Ca^2+^ and Mg^2+^ (Lonza) for 30 min in the dark. Then, the cells were washed twice and incubated with oxidant or HBSS. After incubation, the cells were washed twice, and the fluorescence intensity of 2′,7′-dichlorofluorescein (DCF) was measured, with the excitation and emission set at 485/20 nm and 528/20 nm, respectively, using a Synergy HT spectrophotometer (BioTek Instruments, Winooski, VT, USA).

### 2.7. DNA Damage

The induction of DNA damage was analysed using comet assay [21,22,23]. After treatment, the cells were resuspended in 0.75% low-melting-point agarose and spread onto microscope slides pre-coated with 0.5% normal-melting-point agarose. The slides were immediately put on ice, and the cells were lysed in ice-cold lysis buffer (2.5 M NaCl, 100 mM EDTA, 1% Triton X-100, 10 mM Tris, pH 10) for 1 h. The slides were incubated in an ice-cold developing solution (300 mM NaOH, 1 mM EDTA, pH > 13) for 20 min, followed by electrophoresis in ice-cold electrophoresis solution (30 mM NaOH, 1 mM EDTA, pH > 13) for 20 min at an electric field strength of 0.73 V/cm (32 mA). Then, the slides were washed with H_2_O and stained with 4 µg/mL DAPI. The comets were observed at 200× magnification in an Eclipse fluorescence microscope (Nikon, Tokyo, Japan) attached to a COHU 4910 video camera (Cohu, San Diego, CA, USA) equipped with a UV-1 filter block (an excitation filter of 359 nm and a barrier filter of 461 nm) and connected to a personal computer-based image-analysis system Lucia-Comet version 4.51 (Laboratory Imaging, Prague, Czech Republic). One hundred comets were randomly selected from each sample, and the percentage of DNA in the tail (tail DNA (%)) was measured.

### 2.8. Autophagy Detection with the LC3-II Assay

LC3-II flow cytometry analysis was carried out in accordance with the manufacturer’s protocol. Following the treatment, the cells were washed and stained for autophagic vacuoles using the Autophagy LC3-antibody-based kit (Millipore). Briefly, after the indicated treatment, the cells were incubated with Autophagy Reagent A in EBSS for 5 h at 37 °C to prevent autophagosome-associated LC3 (LC3-II) from degradation while washing away cytosolic LC3 (LC3-I). Then, the cells were washed with ice-cold HBSS and stained with anti-LC3 Alexa Fluor^®^555 in 1× Autophagy Reagent B on ice for 30 min in the dark. Next, the excess of the dye was washed out with ice cold 1× Assay Buffer, and samples were quantified by Muse Cell Analyzer (Millipore). The assay allows for the determination of the Autophagy Induction Ratio (test sample fluorescence relative to control) with the software MuseSoft 1.4.0.0 (Millipore).

### 2.9. Apoptosis

According to the manufacturer’s instructions, apoptosis was examined using the Annexin V and Dead Cell kit (Millipore). Briefly, after the indicated treatment, the cells were incubated with Annexin V and Dead Cell Reagent (7-AAD) for 20 min at room temperature in the dark. The dead, late-apoptotic, early-apoptotic, and live cell events were counted with the Muse Cell Analyzer (Millipore) and analysed with MuseSoft 1.4.0.0 (Millipore).

Caspase activity was quantified by the Muse MultiCaspase assay kit, which permits the simultaneous detection of the presence of multiple caspases (caspase-1 and 3–9). Briefly, after the indicated treatment, the cells were incubated with Muse MultiCaspase Reagent working solution in 1× Caspase Buffer for 30 min at 37 °C in the dark. The Muse MultiCaspase Reagent contains a derivative VAD-peptide, which binds to activated caspases, resulting in the fluorescent signal proportional to the number of active caspases. Then, the cells were counterstained with Muse Caspase 7-AAD working solution, and the events for necrotic cells, dead cells with caspase activity, viable cells exhibiting caspase activity and viable cells without caspase activity were counted with the Muse Cell Analyzer (Millipore) and analysed with MuseSoft 1.4.0.0 (Millipore).

### 2.10. Cell Cycle

After 24 h treatment with oxidant, the cells were collected, washed twice with PBS, resuspended in PBS to a final concentration of 10^6^ cells/mL and allowed to cool for 15 min on ice. One volume of −20 °C absolute ethanol was added to each sample, and the samples were stored at 4 °C until analysis. At that time, the cells were pelleted (400× *g*, 20 min) and resuspended in staining solution containing 50 µg/mL propidium iodide (81845, Sigma-Aldrich) and 50 U/mL RNase A (70856, Merck, Darmstadt, Germany) in PBS. Samples were incubated at 37 °C for at least 30 min in the dark prior to analysis by fluorescence-activated cell sorting (FACS) performed on the LSRII flow cytometer (Becton Dickinson, San Jose, CA, USA) equipped with 488 nm laser excitation and BD FACS Diva software v4.1.2. Data were analysed in FlowJo v10 software (FlowJo LLC, Ashland, OR, USA).

### 2.11. Data Analysis

Statistical analyses were conducted using GraphPad Prism 5 Software (GraphPad Software, San Diego, CA, USA). Statistical comparisons were performed using a Kruskal–Wallis one-way ANOVA multiple comparisons with mean ranks test. Data are expressed as a median ± 95% confidence interval (CI), ***—*p* < 0.001, **—*p* < 0.01, *—*p* < 0.05.

## 3. Results

### 3.1. Induction of G0-like Cell Cycle Arrest, Cell Cycle Reinitiation and Subsequent Arrest in Early G1

**Figure 2:**
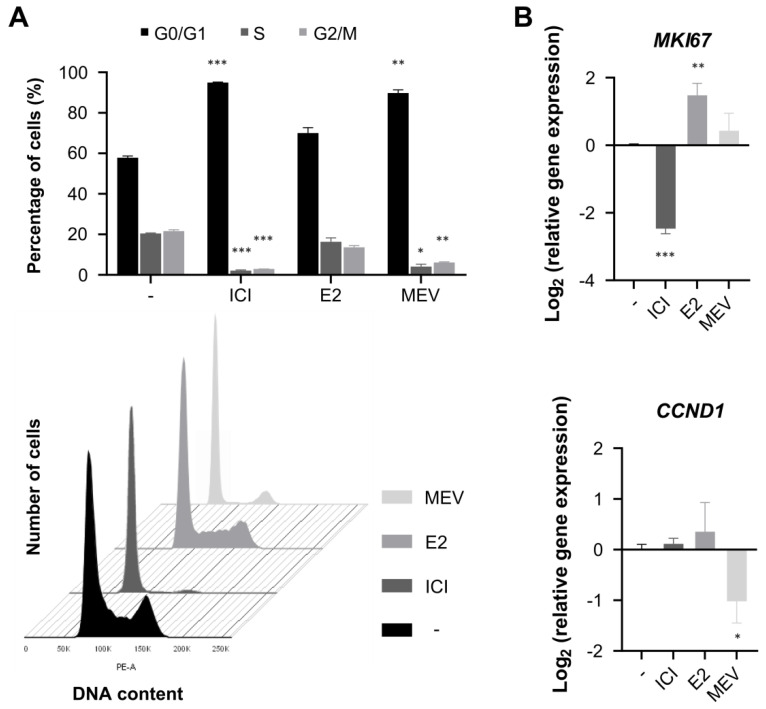
Effect of cell cycle modulation in MCF-7 cells. Arrest in G0-like phase by ICI 182780/fulvestrant (ICI; 10 nM), cell cycle reinitiation by 17β-estradiol (E2; 100 nM) and subsequent cell cycle arrest in G1 by mevinolin (MEV; 40 µM) in MCF-7 cells. (**A**) Cell cycle analysis with FACS histograms of cell cycle profiles (*n* = 9). (**B**) Gene expression was analysed with real-time qPCR and normalised to GAPDH (*n* = 9). Results are presented as median ± CI, *** — *p* < 0.001, ** — *p* < 0.01, * — *p* < 0.05.

To assess the impact of cell cycle modulators in MCF-7 cells, we quantified the DNA content and the expression of *MKI67* and *CCND1* genes. *MKI67* is highly expressed in cycling cells but strongly down-regulated in G0 cells. *CCND1* gene encodes the cyclin D1 protein, an early indicator of G0 → G1 transition. The incubation of exponentially growing MCF-7 cells with ICI increased the population of cells in G0/G1 from 58% to 95% and significantly reduced the expression of *MKI67* (Figure 2 and Figure S2A,B), indicating G0-like cell cycle arrest. Stimulation of the G0-like cells with E2 resulted in a reinitiation of the cell cycle. After 24 h, the cells entered the S phase, and after 48 h, we observed a decrease in the G0/G1 cell population from 95% to 70% with a concomitant increase in cell population in S (16% vs. 2%) and G2/M (14% vs. 3%) and the expression of *MKI67*. E2 induced a fast (6 h, with a maximum at 9 h; Figure S1) increase in the expression of *CCND1*. To arrest cells in G1, the cells, after stimulation with E2, were treated with MEV. MEV-treated cells accumulated in G0/G1 (90%), but contrary to ICI-treated cells, maintained the expression of *MKI67* typical for the cycling cells and the down-regulated expression of *CCND1,* indicating G1 arrest. None of ICI, E2 and MEV affected the viability of MCF-7 cells assessed by trypan blue staining (viability above 93%).

### 3.2. Oxidative Stress Differentially Affects Intracellular ROS Levels in Cells Arrested in the Cell Cycle and Cells That Reinitiated the Cell Cycle

**Figure 3:**
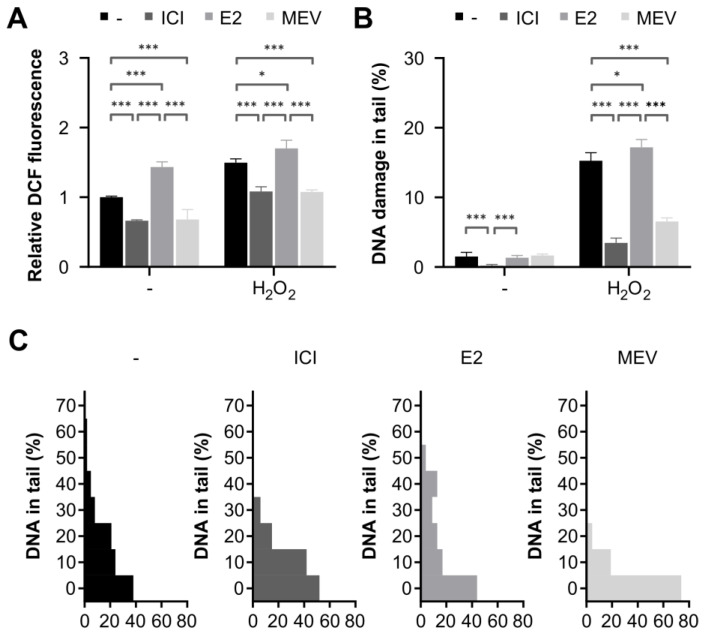
Intracellular reactive oxygen species (ROS) and DNA damage in the G0-like (ICI) cells, the cells that reinitiate cell cycle (E2) and the G1-arrested (MEV) cells exposed to hydrogen peroxide (H_2_O_2_). (**A**) Intracellular ROS level was expressed as the fluorescence of 2′,7′-dichlorofluorescein (DCF) normalised to control cells (*n* = 12). (**B**) DNA damage was measured as the percentage of DNA in the comet tail in the alkaline version of the comet assay (*n* = 200). (**C**) Fraction histograms of DNA in comet tail (*n* = 200) in cells treated with H_2_O_2_. Results are presented as median ± CI, *** — *p* < 0.001, * — *p* < 0.05.

Based on a previous study showing that the levels of ROS are associated with cell cycle progression [24], we evaluated the intracellular ROS levels by measuring DCF fluorescence in the three groups of cells with different cell cycle statuses, namely, G0-like arrested cells, cells that reinitiate cell cycle after G0-like arrest and cells arrested in early G1 with a past incident of G0-like arrest. As summarised in Figure 3, we observed a clear decrease in intracellular ROS levels in the G0-like- and G1-arrested cells (1.5- and 1.4-fold, respectively) and an increase in the cells that reinitiated the cell cycle (1.4-fold). To expand on this observation, we analysed the impact of oxidative stress, induced by H_2_O_2_, on the G0-like- and G1-arrested cells and on the cells that reinitiated the cell cycle. We found that the intracellular ROS level was, likewise, lower in the G0-like- and G1-arrested cells and higher in the cells that re-initiated the cell cycle than in the cells with an unperturbed cell cycle (Figure 3 and Figure S2C). Therefore, these findings imply that the G0-like- and G1-arrested cells effectively lowered, whereas the cells that reinitiate the cell cycle raised intracellular ROS levels in normal and oxidative-stress conditions.

### 3.3. Cells Arrested in The Cell Cycle Are Less Sensitive, and Cells That Reinitiated the Cell Cycle Are More Sensitive to Oxidative-Stress-Induced DNA Damage Than Normal Cycling Cells

The imbalance between ROS production and inactivation could lead to DNA damage and, in consequence, to genomic instability. To determine whether the cell cycle status influences its sensitivity to DNA damage induction, we used the alkaline comet assay and analysed DNA migration during electrophoresis as an estimate of DNA fragmentation. Figure 3B shows a remarkable decrease in DNA fragmentation in the G0-like- (4.4-fold) and the G1-arrested (2.3-fold) cells and an increase in the cells that reinitiated the cell cycle (1.1-fold), compared to the cells with an unperturbed cell cycle under oxidative stress (see also Figure S2D). This finding agrees well with intracellular ROS data (Figure 3A). These results suggest that ROS production rapidly decreases in response to oxidative stress and likely contributes to protection against DNA damage in the G0-like- and the G1-arrested cells. However, in the cells that reinitiate the cell cycle, the augmentation of ROS production promotes the further induction of DNA damage in oxidative stress.

### 3.4. Cells Arrested in the Cell Cycle Are More Resistant, and Cells That Reinitiated the Cell Cycle Are Less Resistant to Oxidative-Stress-Induced Apoptosis Than Normal Cycling Cells

**Figure 4:**
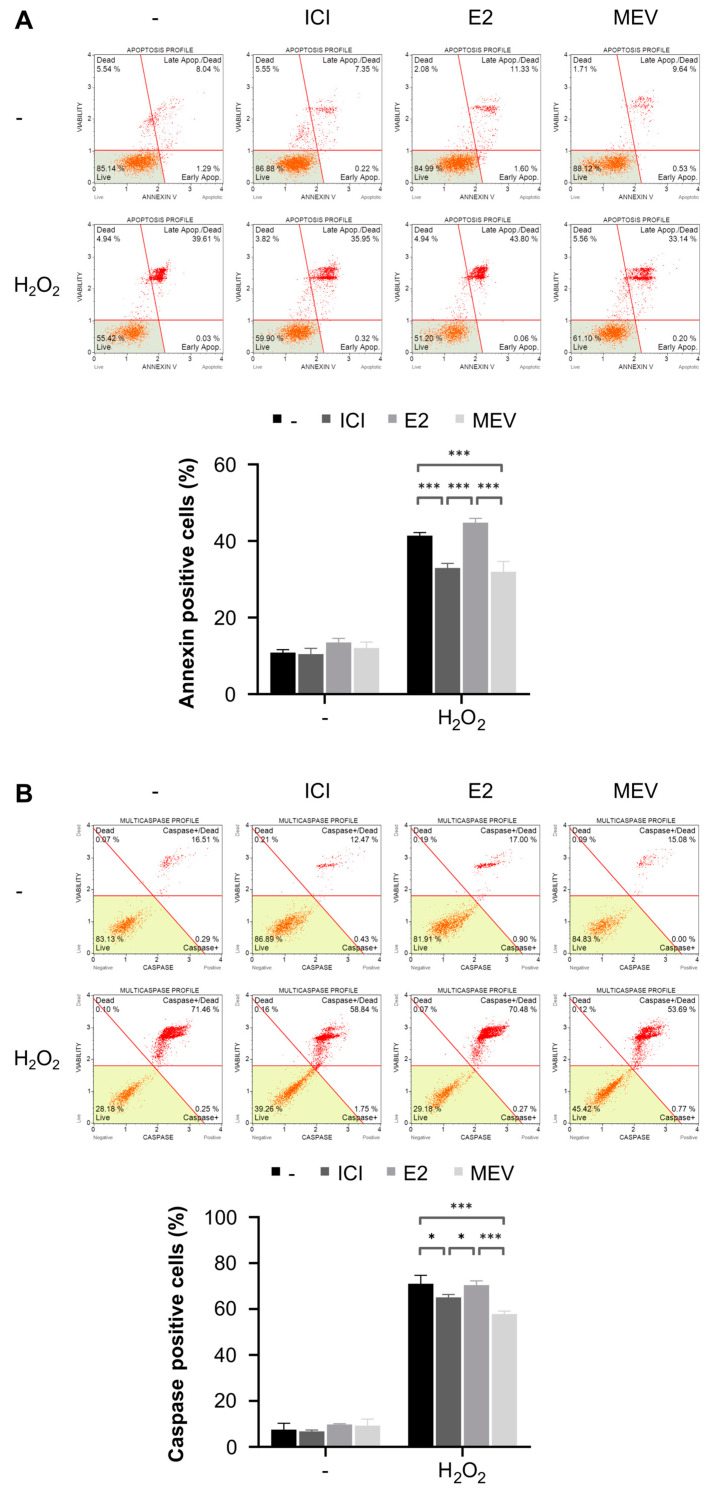
Apoptosis in the G0-like-cells (ICI), the cells that reinitiate cell cycle (E2), and the G1-arrested (MEV) MCF-7 cells exposed to hydrogen peroxide (H_2_O_2_). (**A**) Annexin V externalisation with representative FACS dot plots (*n* = 9). (**B**) Caspase activation with representative FACS dot plots (*n* = 9). Results are presented as median ± CI, *** — *p* < 0.001, * — *p* < 0.05.

The observed differences in intracellular ROS and DNA damage levels between cell groups led us to investigate the sensitivity to cell death in the G0-like- and the G1-arrested cells and the cells that reinitiated the cell cycle in response to oxidative stress. To study this, we stressed the cells with hydrogen peroxide and quantified cell death 4 h later. We found that the G0-like- and the G1-arrested cells significantly reduced, whereas the cells that reinitiated cell cycle increased apoptotic cells (Figure 4 and Figure S2E,F). Figure 4A shows a decrease in late apoptosis, defined by annexin-V^+^/7-AAD^+^, in the G0-like- (1.3-fold) and the G1-arrested (1.3-fold) cells and an increase in late apoptosis in the cells that reinitiated their cell cycle (1.1-fold) compared with the cells with an unperturbed cell cycle. In contrast, each cell group demonstrated a low fraction of early apoptotic cells annexin-V^+^/7-AAD^-^ (below 0.2% of the total population) and a population of necrotic cell annexin-V^-^/7-AAD^+^ population (approx. 11% of the total population).

To confirm these results, each cell group was further investigated for activation of caspase-1 and 3-9, which are apoptosis executive proteins (Figure 4B). Consistent with the reduced annexin-V^+^/7-AAD^+^, the G0-like- and the G1-arrested cells demonstrated fewer caspase^+^/7-AAD^+^ cells (1.2- and 1.1-fold, respectively) compared with the cells with an unperturbed cell cycle in response to oxidative stress (Figure 4B). Similarly to annexin V staining, the population of caspase^+^/7-AAD^+^ was slightly higher, but statistically insignificant, in the cells that reinitiated cell cycle compared to the cells with an unperturbed cell cycle in response to oxidative stress. In each cell group, caspase^+^/7-AAD^-^ and caspase^−^/7-AAD^+^ were of minor importance and constituted below 2% of the total population. The basal frequency of annexin-V^+^/7-AAD^+^ in all groups under normal conditions was approx. 12% and caspase^+^/7-AAD^+^ approx. 7%. These data suggest that decreased intracellular ROS followed by reduced DNA damage in the G0-like- and the G1-arrested cells may protect these cells from oxidative-stress-induced apoptosis and that the augmentation of ROS followed by the increase in DNA damage can favour oxidative-stress-induced apoptosis over survival in the cells that reinitiated cell cycle.

### 3.5. Cells Arrested in the Cell Cycle Increase, and the Cells That Reinitiated the Cell Cycle Decreased Oxidative-Stress-Induced Autophagy Than Normal Cycling Cells

**Figure 5:**
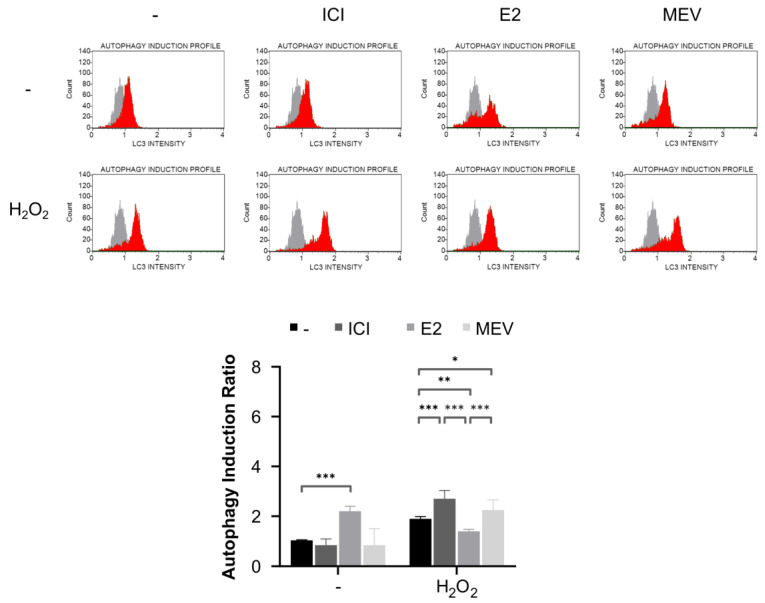
Autophagy in the G0-like-cells (ICI), the cells that reinitiate the cell cycle (E2) and the G1-arrested (MEV) MCF-7 cells exposed to hydrogen peroxide (H_2_O_2_). Autophagy was assessed by analysing the level of autophagosome-associated LC3-II normalised to untreated cells with representative FACS histograms (*n* = 9). Autophagy induction ratio (test sample fluorescence, red histogram, versus control sample fluorescence, gray histogram) is presented. Results are presented as median ± CI, ***: *p* < 0.001, **: *p* < 0.01, *: *p* < 0.05.

Earlier findings indicate that autophagy can be a double-edged sword in response to oxidative stress [25,26]. Primarily, autophagy acts as a cytoprotective mechanism by increasing cellular recycling to neutralise cytotoxic effects. On the other hand, autophagic cell death occurs as a non-apoptotic cell suicide mechanism against cellular stressors in some cases. To investigate whether autophagy was modulated in MCF-7 cells in different cell cycle statuses, we examined the autophagosome-associated LC3-II, a recognised autophagosome marker, in the presence of a lysosome inhibitor reflecting autophagic flux. The G0-like- and the G1-arrested cells strongly upregulated (1.4-fold and 1.2-fold) the LC3-II 4 hr after oxidative stress induction (Figure 5 and Figure S2G). These data are consistent with apoptotic data and suggest that the G0-like- and the G1-arrested cells increase autophagic flux, protecting them against oxidative stress. In support of this hypothesis, the cells that reinitiated the cell cycle less effectively (0.7-fold) activated autophagy under oxidative stress, which is associated with increased susceptibility to apoptosis in these conditions (Figure 4 and Figure 5). Under normal conditions, the cells that reinitiated the cell cycle demonstrated increased autophagic flux compared to control, suggesting that these cells are under some stress. Indeed, cells that reinitiated the cell cycle showed increased intracellular ROS (Figure 3A). The amounts of LC3-II were largely unaffected in the G0-like- and the G1-arrested cells under normal conditions. Collectively, these results show that the G0-like- and the G1-arrested cells increased, and the cells that reinitiated the cell cycle decreased autophagy, in response to oxidative stress.

### 3.6. Oxidative Stress Affects Cell Cycle Status in the Cells That Reinitiated the Cell Cycle and Does Not Affect the Cells Arrested in the Cell Cycle

**Figure 6:**
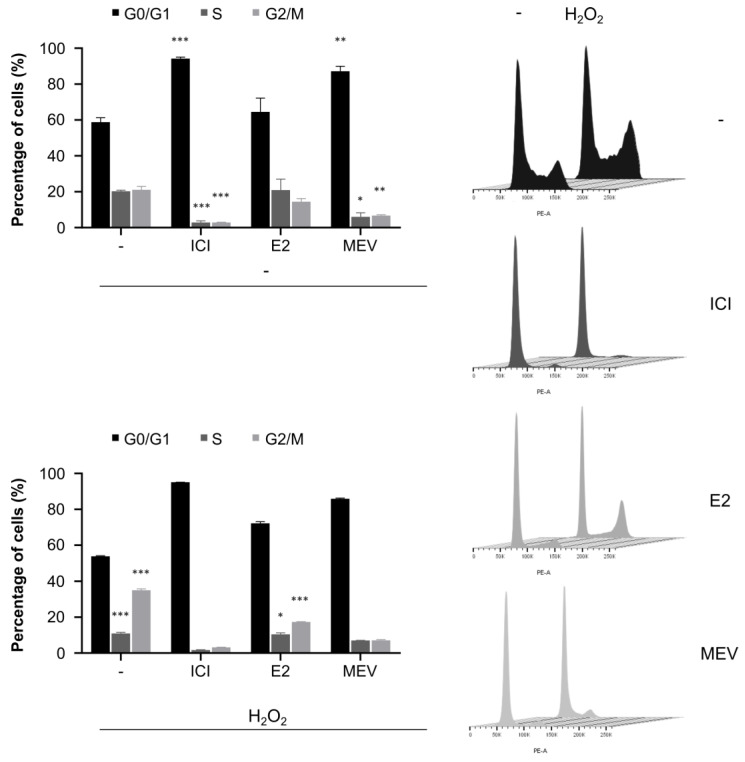
Cell cycle in the G0-like-cells (ICI), the cells that reinitiate the cell cycle (E2) and the G1-arrested (MEV) MCF-7 cells exposed to hydrogen peroxide (H_2_O_2_). Cell cycle profiles with representative FACS histograms (*n* = 9). Statistical comparisons in the upper plot were conducted to non-treated cells (-/-) and in the lower plot to respective cell groups not treated with H_2_O_2._ Results are presented as median ± CI, ***: *p* < 0.001, **: *p* < 0.01, *: *p* < 0.05.

Since DNA damage can stimulate the cell cycle checkpoint activation and/or cell cycle re-entry, thus we analysed the cell cycle under oxidative stress in the cells with different cell cycle statuses by determining DNA content assessed by propidium iodide staining followed by FACS measurement. The oxidant increased the population of G2/M with a concomitant decrease in the S phase in the cells non-treated with cell cycle modulators indicating activation of the G2/M checkpoint, which prevents divisions of cells with damaged DNA (Figure 6 and Figure S2A). The cells that reinitiated the cell cycle demonstrated similar behaviour to those with the unperturbed cell cycle. Both the G0-like- and the G1-arrested cells were largely unaffected by oxidative stress and were unable to progress to G1 and S phases, respectively, under oxidative stress.

## 4. Discussion

Here, we reported a comparative study of some of the functional aspects of DDR in cells at specific cell cycle states, including the arrested cells with features of G0 quiescence (G0-like cells), the cells that reinitiated cell cycle and the cells that were arrested in G1 after G0 exit (G1 cells). We employed a model to manipulate the cell cycle created and developed by Carrol et al., Prall et al., and Rao et al. in cells responsive to oestrogens, which allowed us to study DDR during these specific states [14,15,18]. We investigated the DDR in unperturbed homeostasis and under genotoxic insults induced by oxidative stress. We found that the enforced G0-like phase largely protected the cells from oxidative stress-induced death. Our data showed a strong correlation between apoptosis, ROS production and DNA damage. Some mechanisms appear to protect quiescent cells from oxidative stress, such as the activation of antioxidant defence, low metabolic activity and anaerobic glycolysis [27,28,29,30,31]. In our study, we observed that the protection of G0-like-arrested cells against oxidative stress could be at least partially attributed to increased autophagy. In most, if not all, quiescent cells, metabolism is enormously slowed down, and thus autophagy could function as a mean to provide nutrients for survival [32]. Moreover, the preferential anaerobic glycolysis instead of oxidative phosphorylation of ATP production, reduced biosynthesis and chromatin compaction in quiescent cells can play a role in protection of DNA from damage [28,31,32,33,34]. Indeed, we observed that the G0-like-arrested cells demonstrated significantly reduced DNA damage when compared with control, and under normal (7.8-fold) and oxidative stress (4.4-fold) conditions. In addition, decreased intracellular ROS levels could participate in the protection against DNA damage in quiescent cells.

ROS augmentation might result in cell apoptosis, differentiation or senescence—all processes diminishing the proliferative capacity of temporary cell-cycle-arrested cells such as quiescent cells. The exit from G0-like arrest into the cycle increased intracellular ROS, evoked more DNA damage and sensitised cells that reinitiated the cell cycle to oxidative stress-induced apoptosis. Our data, therefore, support a model in which quiescent cells are protected from apoptosis, and cells that exit quiescence and enter the cycle are more susceptible to death when faced with oxidative stress. Importantly, a lack of resistance to oxidative stress-mediated apoptosis in proliferating cells (including cells that reinitiated the cell cycle) is an organismal safeguard mechanism which eliminates potentially hazardous, genetically unstable cells from an organism, preventing the accumulation of cells susceptible to cancer transformation [35]. After the insult, dead cells can be replaced by neighbouring long-lived quiescent cells, constituting a pool with a regenerative potential [36]. To prevent the depletion of this reservoir, quiescent cells are guarded against DNA replication errors and damage associated with metabolic stress. Eliminating proliferating cells susceptible to cancer transformation, together with the protective and tissue-regenerative potential of quiescent cells, help to maintain tissue homeostasis during oxidative stress insult. To elucidate the relationship between cell cycle withdrawal and the promotion of cell survival under oxidative stress, we studied DDR in cells that followed cell cycle reinitiation and were arrested in early G1. Re-entry into G1 acted as a protective mechanism ensuring the survival of cells in response to oxidative stress. The increased resistance to apoptosis, together with ROS production and DNA damage, was similar to that observed in the G0-like phase under oxidative stress. Correspondingly, we identified a robust activation of autophagic flux in the G1-arrested cells under oxidative stress, similar to that observed in the G0-like-arrested cells. Together with limited autophagic flux in the cells that reinitiated the cell cycle with consequences of cell death under oxidative stress, we suggest that induction of autophagy could be a general protective mechanism for cells arrested in the cell cycle regardless of G0 or G1 phase, and that cell cycle control mechanisms could regulate autophagic flux. The role of autophagy in breast cancer stem cells has recently been demonstrated [10,12]. The inhibition of autophagy impaired the survival of dormant, but not proliferating, BC cells in vitro and in vivo, through the induction of mitochondrial damage and oxidative stress that triggered apoptotic cell death [12]. The inhibition of autophagy was sufficient to reverse chemoresistance in human triple-negative BC stem cells [10]. All the above indicate a crucial role of autophagy in the G0- and the G1-arrested cells. It is likely that not solely autophagy but other fundamental mechanisms of cellular detoxification contribute to the enhanced survival of quiescent and G1-arrested cells.

Our analysis demonstrates that high ROS production led to the extensive accumulation of oxidative stress-induced DNA damage in the cells that reinitiated the cell cycle but not in the G0-like- nor the G1-arrested cells. Here, we have provided evidence that G1/S, but not G0/G1, transition is required to restore an apoptotic response to oxidative stress completely. A possible explanation is that cells entering the S phase conduct DNA replication, which creates an opportunity to induce DNA damage and secondary lesions that can activate apoptotic signalling [37,38]. In contrast, repairable DNA damage requires a progression from G0 to early G1 [39]. Multiple pieces of evidence, together with our own, suggest that DNA damage eventually evoking cell death may require the cell to enter the S phase and conduct DNA replication in addition to G0/G1 transition [37,38,39].

Applying chemical compounds to modulate the cell cycle in this study proved to be highly effective in arresting the cells in the G0-like phase, inducing cell cycle reinitiation, and arresting the cells in G1. However, the chemicals employed for cell cycle manipulation could affect other pathways, primarily oestrogen signalling and cholesterol synthesis, which could also affect DDR. One limitation of the study is that the long-term treatment with oestrogens induced DNA damage-activated DDR signalling [40,41]. Thus, we cannot exclude the possibility that E2, apart from the induction of cell cycle reinitiation, could influence DDR pathways, especially since we observed an increased level of intracellular ROS and autophagy flux after E2 treatment. Moreover, extending the research to other BC cell lines would allow for the generalisation of our findings. However, scarce research on cell cycle modulation via oestrogen signalling has been conducted on other than MCF-7 oestrogen receptor (ER)-positive BC cell lines. We believe that further studies on DDR signalling pathways, including autophagy, in the cell cycle context should shed more light on the role of the cell cycle orchestration of DDR.

## 5. Conclusions

Our present study provides a novel mechanistic insight into the response to oxidative stress in a cell cycle-dependent manner. Cell cycle state dictates the fate of cells insulted by oxidative stress: G0-like- and G1-arrested cells are predisposed to survival, whereas cells that reinitiated the cell cycle are predisposed to death. We identified autophagy as one explanation for the observed effect, which depends on the cell cycle status. We believe that undertaking studies on the role of the cell cycle in DDR is essential from the perspective of drugs targeting diseases in which cell cycle progression is disturbed.

## Data Availability

The data presented in this study are available on request from the corresponding author.

## Supplementary Materials

The following supporting information can be downloaded at: https://www.mdpi.com/article/10.3390/cimb45080399/s1.
