# Supplementary material for: Cell Cycle Status Influences Resistance to Apoptosis Induced by Oxidative Stress in Human Breast Cancer Cells, Which Is Accompanied by Modulation of Autophagy
Source: Curr Issues Mol Biol. 2023 Jul 29;45(8):6325–38. doi: 10.3390/cimb45080399 (PMC10453102; doi:10.3390/cimb45080399)
Supplement: Supplementary file 1 [file cimb-45-00399-s001.zip › cimb-2487058-supplementary.pdf]

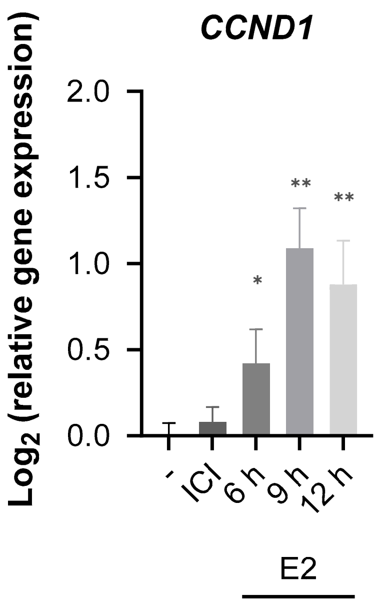

Figure S1. Effect of cell cycle modulation in MCF-7 cells. Gene expression in MCF-7 cells arrested in G0-like phase by ICI 182780/fulvestrant (ICI; 10 nM) and cells which reinitiated cell cycle by 17β-estradiol (E2; 100 nM). Gene expression was analysed with real-time qPCR and normalised to GAPDH (n = 9). Results are presented as median ± CI, ** – $p < 0.01$, * – $p < 0.05$.

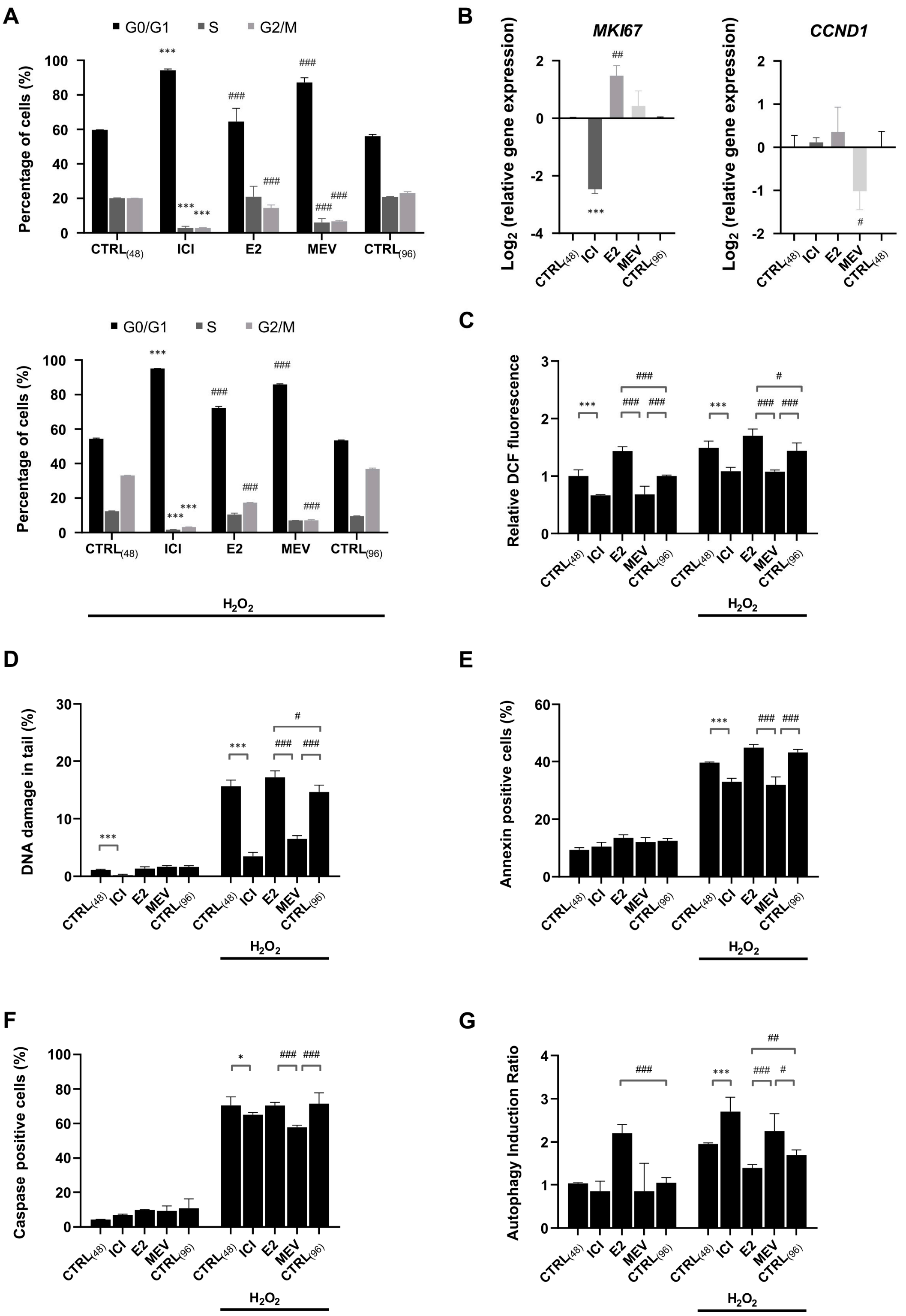

Figure S2. DNA damage response in the G0-like- (ICI), the cells that reinitiate cell cycle (E2) and the G1-arrested (MEV) MCF-7 cells exposed to hydrogen peroxide (H_2O_2). (A) cell cycle modulation, (B) gene expression, (C) intracellular reactive oxygen species (ROS), (D) DNA damage, (E) apoptosis assayed as annexin V externalisation, (F) caspase activation, (G) autophagy assayed as the level of autophagosome-associated LC3-II. Negative controls for different exposure times: 48 h ($CTRL_{(48)}$) for ICI treatment and 96 h ($CTRL_{(96)}$) for E2 and MEV treatments are shown. Statistical comparisons were conducted to $CTRL_{(48)}$ (*) for ICI treatment or to $CTRL_{(96)}$ (#) for E2 and MEV treatment.
